# Evaluation of the Safety and Efficacy of Transcatheter Closure of Perimembranous Ventricular Septal Defects with a Single Device Type: A Single-Centre Experience

**DOI:** 10.3390/jcm14217822

**Published:** 2025-11-04

**Authors:** Piotr Weryński, Oksana Trębacz, Wojciech Tarała, Patrycja Florek, Jacek Podlewski, Robert Sabiniewicz

**Affiliations:** 1 Department of Pediatrics and Pediatric Gastroenterology with Pediatric Cardiology Subdivision, St. Jadwiga the Queen Clinical Regional Hospital No. 2, 35-301 Rzeszów, Poland; 2 Department of Neurobiology, Maj Institute of Pharmacology, Polish Academy of Science, 31-343 Krakow, Poland; 3 Dover Fueling Solutions, 30-320 Krakow, Poland; 4 Department of Pediatric Cardiology and Congenital Heart Diseases, Medical University of Gdansk, 80-211 Gdansk, Poland

**Keywords:** perimembranous ventricular septal defect, transcatheter closure, young children, Nit-Occlud^®^ Lê VSD Coil

## Abstract

**Background/Objective**: Ventricular septal defect (VSD) is the most common congenital heart anomaly, with the perimembranous subtype (pmVSD) being among the most prevalent forms. Surgical repair remains the gold standard for treatment; however, percutaneous closure has emerged as a promising alternative due to the availability of various occlusion devices. Each technique presents distinct advantages and limitations, particularly in terms of complications and long-term outcomes. We sought to evaluate the safety and mid-term outcomes of pmVSD closure employing the Nit-Occlud^®^ Le VSD Coil within a single-centre, single-team setting. **Methods**: Of the 55 patients hospitalised for pmVSD closure, 45 children underwent the procedure with the Nit-Occlud^®^ Le VSD device. Relevant clinical and defect-related data were collected during hospitalisation and throughout follow-up. **Results**: Among 45 patients, successful coil implantation was achieved in 41 cases (91.1%). Periprocedural complications occurred in 8 patients (17.8%), including haemolysis, transient atrioventricular block, aortic valve injury, transient ST-segment elevation and supraventricular tachycardia. Two of these complications (4.4%) were classified as severe. The occurrence of complications was significantly associated with the type of VSD shunt (*p* = 0.03). **Conclusions**: Transcatheter closure of pmVSD using the Nit-Occlud^®^ Lê VSD Coil in young children is a feasible and safe option with careful patient selection. Patients with type C pmVSD appear to benefit the most. Nevertheless, potential complications, including haemolysis and aortic valve injury, require close monitoring.

## 1. Introduction

Ventricular septal defect (VSD) is the most prevalent congenital cardiac malformation, accounting for up to 40% of cases. Among them, perimembranous defects (pmVSDs) are one of the most common subtypes [1,2]. They are located at the anteroseptal commissure behind the septal leaflet of the tricuspid valve and below the commissure between the right and noncoronary leaflets of the aortic valve, in close proximity to the atrioventricular (AV) conduction system [3]. Consequently, VSD closure carries the risk of damaging surrounding structures, including valve injury and complete AV block. For around 70 years, direct surgical closure of the defect has been regarded as the standard treatment, starting with Lillehei and colleagues performing the first VSD closure in 1955 [4,5]. On the other hand, over the past twenty years, transcatheter techniques for VSD closure have been developed. However, in the case of pmVSDs, significant concerns persist, particularly regarding the risk of post-procedural and late-onset AV block. This complication has been notably associated with earlier-generation asymmetric devices, with reported incidence rates ranging from 2.9% to 5.7% in various studies [6,7,8]. As a result, modified devices that decrease the radial force on the defect margins and remove the clamp force have been used for pmVSD closure, yielding results similar to surgery [9]. In line with these findings, Sivakumar et al. reported no cases of AV block following transcatheter pmVSD closure in their patient group, regardless of whether dedicated or off-label devices were utilised [10].

Given the wide range of VSD closure devices, the Nit-Occlud^®^ Le VSD Coil (PMF, Schweix, Germany) appears to be an effective and safe option for pmVSD closure. Its low profile and retrievable design contribute to a favourable safety profile, with no reported cases of complete AV block [11,12,13]. The device’s self-adapting configuration allows it to conform to the shape of the VSD, minimising pressure on the septal margins [14]. Additionally, the distal end is covered with Dacron fibres to facilitate early defect closure. However, some research indicates higher rates of residual shunts and haemolysis, suggesting that the therapeutic strategy with this occluder remains controversial and is limited to select patients [15]. Considering the variability of existing data, we aimed to evaluate both safety, defined as the incidence of procedure- and device-related complications, and mid-term outcomes, including defect closure success, residual shunt rates, and late-onset complications following pmVSD closure with the Nit-Occlud^®^ Le VSD Coil. This evaluation was conducted in a single-centre, single-team setting, following a standardised, unified protocol for both the closure procedure and the follow-up period.

## 2. Materials and Methods

### 2.1. Study Design

A study was conducted employing an observational, single-centre, single-team cohort design. We included all children diagnosed with pmVSD between June 2022 and August 2025 who were admitted to the Department of Paediatrics and Paediatric Gastroenterology, Paediatric Cardiology Subdivision in Rzeszów, for defect closure utilising the Nit-Occlud^®^ Le VSD Coil system. Demographic data, selected information from patients’ medical records, echocardiographic findings, and hemodynamic examinations were analysed. Patients with significant left ventricular volume overload were eligible for VSD closure. The local ethics committee approved the study (No 26/2025/B) on 2 July 2025. Consequently, most of the data were analysed retrospectively, while a portion was analysed prospectively.

### 2.2. Patient Selection

All patients were evaluated in a paediatric echocardiography laboratory using a Philips EPIQ 7C machine. Left ventricular size was assessed in both the parasternal long-axis and apical four- and five-chamber views. chamber views. Z-scores were calculated using the Detroit Formula [16], where a Z-score of the left ventricular end diastolic diameter ≥2 indicated significant volume overload. Pulmonary artery pressures were estimated based on pulmonary and tricuspid regurgitation. The Doppler gradient across the VSD was measured using continuous-wave Doppler. To classify pmVSD, we adopted the Echocardiographic Classification of pmVSD developed by Sivakumar et al. [10]. This system is based on the relationship of the VSD margins to the aortic and tricuspid valves, as well as the presence of a septal aneurysm. In summary, there are four morphological types of defects: Type A, which lacks a superior VSD margin separating it from the aortic annulus; type B, where a well-developed ventriculo-infundibular fold separates the defect from the aortic valve, creating a muscular aortic margin; type C, where a membranous septum forms an aneurysm; and type D, which includes an aneurysm formed by accessory tricuspid leaflets [17]. In cases involving an aneurysm, there can be more than one leak on the right ventricular end, separated by membranous tissue or tricuspid chords.

### 2.3. VSD Closure Protocol

**Figure 1:**
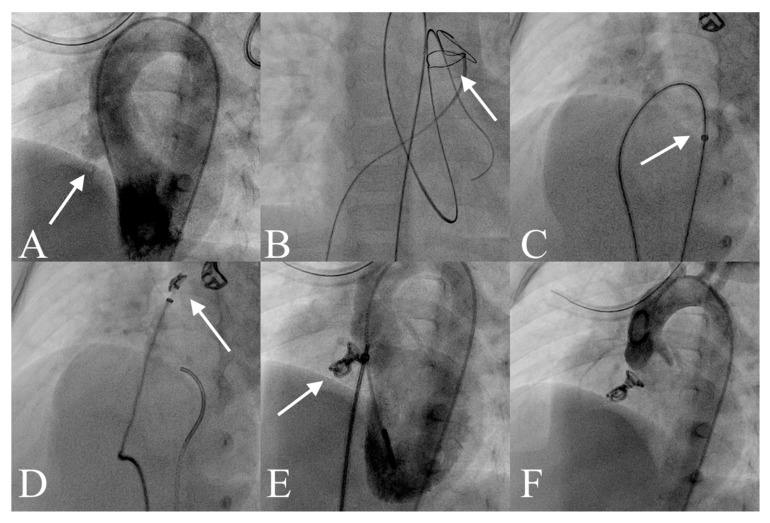
Transcatheter pmVSD Closure with the Nit-Occlud^®^ Le VSD Coil. (**A**) Left ventricular angiography showing a pmVSD with aneurysmal tissue (white arrow). (**B**) A guidewire advanced through the defect from the left ventricle to the right ventricle and into the left pulmonary artery, forming an arteriovenous loop (white arrow). (**C**) The delivery catheter (white arrow) was advanced via venous access into the descending aorta, and the occluder was introduced through the catheter. (**D**) Formation of distal loops (white arrow). (**E**) Left ventricular angiography confirms appropriate device position (white arrow) with minimal residual shunt through the defect. (**F**) Final device position after release, with no evidence of aortic regurgitation. Abbreviations: pmVSD, perimembranous ventricular septal defect.

The transcatheter pmVSD closure procedures were conducted in the catheterisation laboratory under both echocardiographic and fluoroscopic guidance by the same experienced team of operators. All patients underwent the procedure under general anaesthesia. Under ultrasound guidance, the common femoral artery and vein were cannulated using 4F and 5F sheaths, respectively, followed by intravenous administration of heparin at a dose of 75–80 IU/kg. Left ventricular angiography was performed in the left anterior oblique (LAO) view with cranial angulation (LAO 50°, CRA 30°) to assess the size and morphology of the pmVSD (Figure 1A).

Device selection was guided by angiographic measurements, with the distal diameter of the coil chosen to be at least twice the effective diameter of the VSD on the right ventricular side and approximately 1–2 mm larger than the diameter measured from the left ventricular side [18]. After the evaluation, the defect was crossed retrogradely from the left ventricle using a 4F Berenstein catheter and a floppy hydrophilic wire. The wire was then snared on the right side, typically within the pulmonary artery, to establish an arteriovenous (a-v) loop (Figure 1B). The delivery sheath was introduced via the venous access and advanced over the wire across the VSD into the ascending aorta (Figure 1C). The Nit-Occlud^®^ Lê VSD Coil was delivered through the sheath into the ascending aorta, where the distal loops were formed (Figure 1D). Except for the final two loops, all other loops of the coil were deployed in the ascending aorta. Gentle retraction of the system allowed it to cross the aortic valve and position the device across the defect (Figure 1E,F). When the coil was pulled back into the VSD, it typically conformed to the shape of the defect, assuming a conical configuration. Once the deployed loops were securely anchored within the VSD, the remaining two loops were positioned on the right ventricular side of the defect. For type C defects, the device was placed within the aneurysmal tissue to prevent protrusion into the left ventricular outflow tract. In patients without aneurysmal tissue, the coil ends were positioned on the left and right sides of the septum, respectively. The implantation was carried out utilising combined transthoracic echocardiography (TTE) and angiographic guidance.

Collected clinical data included patient demographics, presenting signs and symptoms, and TTE findings, with a detailed assessment of residual shunt and device-related complications. The complications evaluated included AV block or other rhythm and conduction disturbances, device-associated aortic or tricuspid valve injury, haemolysis, device embolisation, endocarditis, and vascular access site complications. All patients underwent follow-up evaluations within 24 h post-procedure, at discharge, and subsequently at 1, 6, and 12 months, followed by annual assessments.

### 2.4. Statistical Analysis

Continuous variables were presented as medians and interquartile ranges (IQR) and compared with the Mann–Whitney U test, as almost all of them had non-normal distributions (assessed with the Shapiro–Wilk test). Categorical variables and ranges were presented as numbers (percentages), and confidence intervals for proportions were calculated using the Clopper–Pearson method. Analysis was performed with R software version 4.4.2. A *p*-value less than 0.05 was considered statistically significant for all tests.

## 3. Results

**Figure 2:**
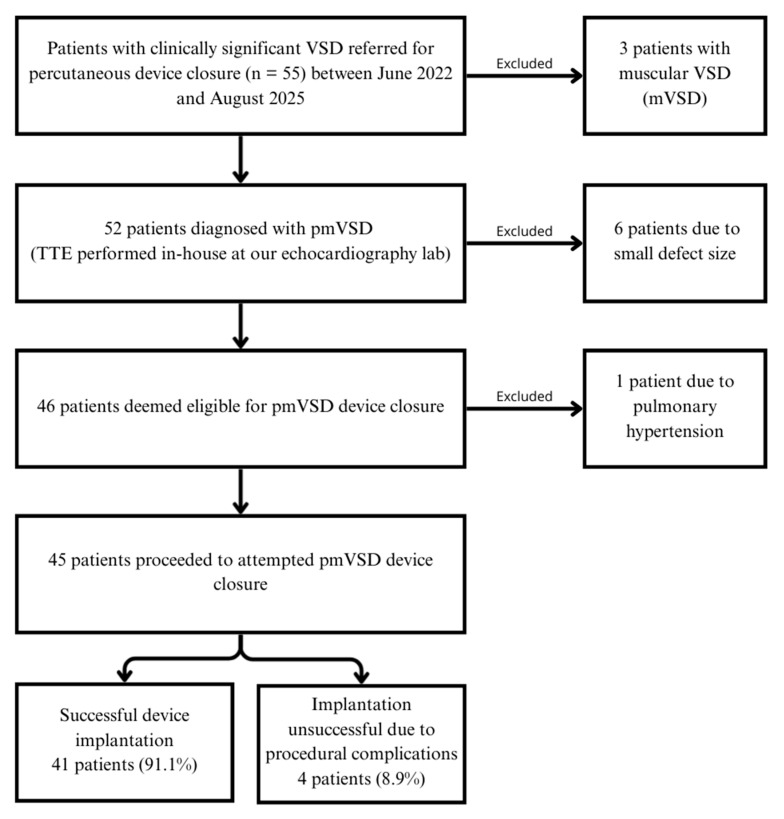
Study flow chart.

During the study period, 55 patients were admitted for VSD closure. Following comprehensive TTE evaluations, three patients were excluded due to muscular VSD, and six others had defects too small to warrant closure. Of the remaining 46 patients, one was excluded due to pulmonary hypertension and was referred for surgical pulmonary artery banding. The patient selection process is outlined in the study flow chart (Figure 2).

Patient and VSD characteristics are summarised in Table 1. Briefly, the study population had a median (interquartile range [IQR]) age of 2.69 years (1.98–4.86). There was a slight predominance of females over males, with 27 (60%) and 18 (40%) patients, respectively. According to the echocardiographic classification [10], 2 patients (4.4%) had type A VSD, 10 patients (22.2%) had type B VSD, and 33 patients (73.3%) had type C VSD. No patients were found to have a type D defect. Most of the VSDs were native, with 43 cases (96%) classified as such, and 2 cases (4%) identified as postoperative residual.

Multiple-exit pmVSD was detected in 13 patients (29%) with type C defects.

The median (IQR) VSD size was 7.2 (4.2–9.5) mm on the left side and 3 (2.5–3.4) mm on the right side. All VSD closures were performed using the Nit-Occlud^®^ Lê VSD Coil system. In the majority of cases, the initially selected device size was appropriate. However, size adjustments were necessary in three patients: two required a larger coil, and one required a smaller one. Table 2 provides an overview of the device sizes utilised.

Abbreviations: see Figure 1; pmVSD, perimembranous ventricular septal defect; TTE, transthoracic echocardiography.

**Figure 3:**
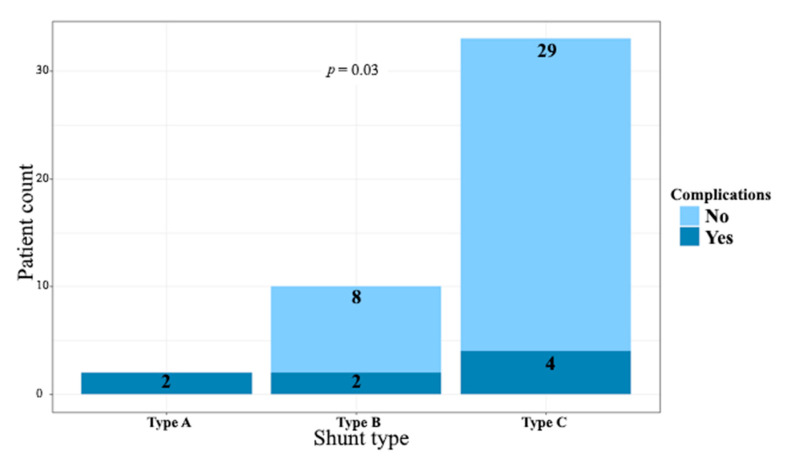
Correlation between pmVSD shunt type and risk of periprocedural complications. Patients with type C pmVSD had a significantly lower risk of complications compared with those with type A or type B defects. Abbreviations: see Figure 1.

Successful coil implantation was achieved in 41 cases (91.1%, 95% confidence interval [CI] 78.8–97.5%). For the remaining four patients, coil implantation was not completed due to procedural complications. A complete AV block was observed in one case after a-v loop formation, but it resolved immediately upon wire retrieval. In one child, the VSD was in close proximity to the aortic valve, preventing full coil expansion due to the risk of interference with the aortic leaflets. Another patient experienced ST-segment elevation, bradycardia, and a drop in blood pressure during the closure attempt; these symptoms resolved immediately following coil retrieval. The fourth patient sustained an iatrogenic injury to the right coronary aortic cusp, resulting in moderate aortic regurgitation, which required surgical aortic valvuloplasty. Furthermore, the aforementioned three patients in whom transcatheter pmVSD closure was unsuccessful were rescheduled for conventional surgical VSD closure. A residual trace shunt was observed in 25 (61%) cases immediately following implantation, decreasing to 17 (42.5%) at discharge and 7 (17.5%) at later follow-up visits. Periprocedural complications occurred in 8 patients (17.8%; 95% CI: 8.0–32.1%), of which two were classified as severe (4.4%; 95% CI: 0.5–15.1%). Aside from the three procedural complications mentioned earlier, which included severe iatrogenic aortic regurgitation, five additional complications were observed. Mechanical haemolysis affected three patients (6.7%), with two cases self-limiting and one severe, resulting in device migration on the fourth day after the procedure and requiring surgical intervention. One patient developed AV block on the second day post-procedure, converting to a left bundle branch block before resolving spontaneously. The final patient experienced supraventricular tachycardia, successfully treated with adenosine. No tricuspid valve injuries or local puncture site complications were observed in our patient cohort. The occurrence of complications was significantly associated with the VSD shunt type (*p* = 0.03). In contrast, residual shunt (*p* = 0.43, Figure 3), multiple-exit VSDs (*p* = 0.60), as well as patient age, weight, and height showed no significant association with complications.

All patients who underwent successful device closure were followed for a median (IQR) period of 41 months (12.3–75.8). No delayed severe complications were observed during follow-up, including endocarditis, haemolysis, aortic or tricuspid valve lesions, AV block, or device embolisation. The clinical success rate was 69% (95% CI 53.4–81.8%), defined as complete closure of the defect with no residual flow and no late complications in mid-term follow-up.

## 4. Discussion

This study represents the largest single-centre, single-team experience with the percutaneous closure of perimembranous ventricular septal defects using the Nit-Occlud^®^ Le VSD Coil in Poland. Our patient cohort was younger than those reported in previous studies [12,15]; however, all were over one year of age, a known risk factor for procedural complications [6,19]. Carminati et al. previously reported that young age and low body weight are associated with an increased risk of early complications following transcatheter pmVSD closure. Consequently, successful interventions in young patients, such as those in our cohort, with a median (IQR) weight of 14.2 kg [12,13,15,16,17,18,19], require low-profile devices compatible with small-calibre delivery systems (6–7 F). The Nit-Occlud^®^ Le VSD Coil system fulfils these criteria and plays a crucial role in reducing the risk of complications in this vulnerable population. Notably, recent reports have described successful pmVSD closures in infants weighing less than 10 kg, using alternative closure platforms [20,21].

Device sizing with the Nit-Occlud^®^ Le VSD Coil device was generally straightforward, with only 3 of 45 patients requiring readjustment. Angiography was used for precise measurements, and coil diameters were selected as outlined in the Section 2. For aneurysmal tissue cases (type C), the device was placed inside the aneurysm sac to reduce contact and friction against the aortic valve.

Successful coil implantation was achieved in 41 cases (91.1%, 95% CI 78.8–97.5%), placing this result in the median range compared to other studies using this type of occluder [12,15]. However, in three patients, the pmVSD was not closed due to periprocedural complications, and in one case, the procedure was abandoned due to unfavourable defect anatomy (type A). Overall, complications occurred in 8 patients (17.8%), a rate comparable to those reported in previous studies [11,12,22], and lower than that reported by others [15]. The main complication observed was mechanical haemolysis, occurring in three patients. In two of these cases, the haemolysis was transient, self-limiting, and resolved with medical therapy alone. However, in one patient, it was severe and ultimately led to device migration. The occluder embolised to the right ventricle on the fourth day after deployment, necessitating surgical intervention. The patient underwent successful cardiac surgery for device removal and VSD closure, with an uneventful recovery. Haemolysis associated with intracardiac prosthetic material is a well-documented complication, particularly following pmVSD closure with various types of occluder devices [23]. This has been reported frequently with the Nit- Nit-Occlud^®^ VSD coil [12,15,22,24]. As noted previously, mechanical haemolysis was more commonly observed in cases with residual shunting, especially when multiple-exit VSDs were present [15]. In our study, all three patients who developed haemolysis had residual shunts following device implantation. However, we did not find a statistically significant link between residual shunting and the occurrence of complications, including haemolysis (*p* = 0.43). This may be explained by the presence of only trace or mild residual shunts, without moderate or hemodynamically significant leakage, which are less likely to cause mechanical haemolysis. Another possible explanation is the limited sample size; in a larger cohort, the association between residual shunting and complication rates might become more apparent. Although the French group [15] studied a similar population and reported a significantly higher incidence of haemolysis, the defects and device sizes in their cohort were larger than in ours. It is therefore reasonable to speculate that the increased shunt size contributed to the higher rate of complications observed in their study. In any case, the majority of studies reported that even higher rates of mechanical haemolysis resolved spontaneously with medical treatment alone, with only isolated cases requiring blood transfusion.

Residual shunting immediately following pmVSD closure was relatively common, observed in 25 patients (61%) post-implantation. This rate declined to 17 patients (42.5%) at the time of discharge and further declined to 7 patients (17.5%) during follow-up. These findings are consistent with previous studies using the same device [11,12,13,25]. In comparison, the incidence of residual shunt after pmVSD transcatheter closure with different occluders has been reported to range from 8.3% to 30.8% [19,26]. Notably, approximately one-third of these residual shunts gradually resolved during follow-up [19,27]. The initially high rate of residual shunting may be attributed to the device’s unique design and occlusion mechanism. Unlike devices with a stenting component, the Nit-Occlud^®^ Coil Le VSD relies on a single coil inserted into the defect for closure. Although polyester fibres are attached on the left ventricular side to promote thrombosis, the absence of a scaffold-like structure may result in a longer time to complete occlusion [12,18].

Another group of complications observed during and after pmVSD closure involves rhythm and conduction disturbances. In our study, one case of complete atrioventricular (AV) block occurred during the attempt to close the defect but resolved immediately after retrieval of the closure system. A second case of complete AV block developed on the second day post-procedure; it was transient and resolved spontaneously. Neither case required medical therapy or pacemaker implantation. Additionally, no late-onset conduction disturbances were observed during follow-up. Our findings are consistent with previous studies. While a complete AV block has been a notable concern with asymmetric devices used for pmVSD closure [7,8], the Nit-Occlud^®^ Coil Le VSD coil is generally considered safe and is not typically associated with persistent conduction disturbances [11,12,13,18]. Transient supraventricular tachycardia, experienced by one patient in our cohort, is a benign and self-limiting arrhythmia that can occur during pmVSD closure procedures. It is well-documented in the literature as a transient event with no long-term clinical impact [19,27].

The final category of complications involved aortic valve-related issues. Specifically, one patient developed ST-segment elevation, bradycardia, and hypotension during the closure attempt while an a–v loop was being formed. This presentation was likely due to severe iatrogenic aortic regurgitation (AR) caused by the temporary immobilisation of an aortic cusp by the a–v loop, leading to acute myocardial ischemia. The condition resolved immediately upon retrieval of the system. Myocardial ischemia caused by severe AR is a well-known complication, most likely due to diastolic steal syndrome, specifically the backflow of blood from the aorta into the left ventricle, which disrupts coronary flow dynamics. It usually resolves completely once the underlying cause, namely the aortic regurgitation, is treated [28]. In some cases, iatrogenic AR may be permanent rather than transient, caused by direct injury to the aortic valve cusps. According to the literature, new-onset AR following transcatheter closure of pmVSD occurs in approximately 3.3–4.5% of patients [6,27]. Notably, the right coronary cusp is most frequently affected, as previously reported with the Nit-Occlud^®^ Coil Le VSD device [15] as well as with other occluder types [27]. Similarly to our observation, these injuries often resulted in moderate AR, requiring aortic valve repair along with surgical closure of the VSD.

To our knowledge, this is the first study to directly associate the risk of complications with the anatomical type of pmVSD. Our findings show a significant correlation between complication rates and shunt type (*p* = 0.03), with patients having type C pmVSD demonstrating a significantly lower risk of complications compared to those with type A or type B defects. However, it is important to note that previous reports have highlighted similar trends. For instance, Nguyen et al. [12] reported higher rates of procedural failure and complications in patients lacking an aortic rim (type A pmVSD, according to the adopted classification). Conversely, Roy et al. [20] observed that patients with aneurysmal tissue (also classified as type C in our study) were more suitable candidates for percutaneous closure.

Contrary to the findings of a previous study [6], we failed to demonstrate a correlation between complication rates and patient age, weight, or height. While the limited sample size in our cohort may have contributed to this discrepancy, it is also noteworthy that the complications reported in the referenced European Registry [6] were predominantly related to the occurrence of complete AV block, which is not a concern when using the Nit-Occlud^®^ Coil Le VSD device.

### Limitations

Several limitations of this study should be acknowledged. Firstly, since all procedures were performed by a single, highly experienced team at one centre, the results may not fully reflect outcomes achievable in routine clinical practice. This single-centre, single-team approach may therefore introduce bias and limit the wider applicability of the findings. Secondly, although this study involved the largest cohort of patients treated with this device in Poland, the sample size remains inadequate for broad generalisation. Another potential source of bias is the partly retrospective design; however, adhering to a strict institutional protocol for percutaneous pmVSD closure helped ensure comprehensive and reliable data collection. Lastly, the mid-term follow-up of 41 months was not sufficient to fully evaluate late complications, particularly the occurrence of complete atrioventricular block.

## 5. Conclusions

Transcatheter closure of pmVSD using the Nit-Occlud^®^ Lê VSD Coil in young children is both feasible and safe when performed by an experienced team, with a low incidence of complete AV block. Optimal outcomes depend on meticulous patient selection, preferably those with type C, medium-sized pmVSD, and precise device deployment within the aneurysmal sac. Nonetheless, potential complications such as aortic valve injury, haemolysis, and device migration warrant close attention.

## Figures and Tables

**Table 1:** Baseline patient and pmVSD characteristics.

All Patients		Without Complications	With Complications	*p*-Value
Age, years, median (IQR)	2.69 (1.98–4.86)	2.84 (2.01–4.91)	2.64 (1.96–3.23)	0.54
Sex, females	18 (40)	14 (37.8)	4 (50)	0.69
Weight, kg, median (IQR)	14.2 (12–18)	14.6 (12–18)	13.85 (12.25–16.5)	0.69
Height, cm, median (IQR)	92 (88–106)	92 (88–106)	92.5 (87.5–100.38)	0.66
pmVSD left side size, mm, median (IQR)	7.2 (4.2–9.5)	7.6 (4.5–9.6)	5.25 (3.83–9.12)	0.33
VSD right side size, mm, median (IQR)	3 (2.5–3.4)	3 (2.5–3.4)	3 (2.63–3.35)	0.96
Shunt type				
Type A	2 (4.4)	0 (0)	2 (25)	0.03
Type B	10 (22.2%)	8 (21.6%)	2 (25%)	
Type C	33 (73.3%)	29 (78.4%)	4 (50%)	
Type D	0 (0%)	0 (0%)	0 (0%)	-
Multiple-exit pmVSD	13 (29.5%)	10 (27.8%)	3 (37.5%)	0.68

Data are presented as numbers (percentages) unless otherwise indicated. Abbreviations: IQR, interquartile range, pmVSD, perimembranous ventricular septal defect.

**Table 2:** Nit-Occlud^®^ Le VSD Coil device sizes in pmVSD closure.

Device Size	Successful Coil Implantation (41 Patients)
8/6	16 (39)
10/6	21 (51.2)
12/6	4 (9.8)

Data are presented as numbers (percentages) unless otherwise indicated. Abbreviations: see Table 1.

## Data Availability

The original contributions presented in this study are included in the article. Further inquiries can be directed to the corresponding author.
